# Impaired function in the lung periphery following COVID‐19 is associated with lingering breathing difficulties

**DOI:** 10.14814/phy2.15918

**Published:** 2024-01-22

**Authors:** Sanna Kjellberg, Alexander Holm, Nicolas Berguerand, Helena Sandén, Linus Schiöler, Monika Fagevik Olsén, Anna‐Carin Olin

**Affiliations:** ^1^ Occupational and Environmental Medicine, School of Public Health and Community Medicine, Institute of Medicine, Sahlgrenska Academy University of Gothenburg Gothenburg Sweden; ^2^ Department of Respiratory Medicine and Allergology Sahlgrenska University Hospital Gothenburg Sweden; ^3^ Department of Health and Rehabilitation/Physiotherapy Institute of Neuroscience and Physiology, Sahlgrenska Academy, University of Gothenburg Gothenburg Sweden

**Keywords:** multiple breath washout, oscillometry, SARS‐CoV‐2

## Abstract

Lingering breathing difficulties are common after COVID‐19. However, the underlying causes remains unclear, with spirometry often being normal. We hypothesized that small airway dysfunction (SAD) can partly explain these symptoms. We examined 48 individuals (32 women, 4 hospitalized in the acute phase) who experienced dyspnea and/or cough in the acute phase and/or aftermath of COVID‐19, and 22 non‐COVID‐19 controls. Time since acute infection was, median (range), 65 (10–131) weeks. We assessed SAD using multiple breath washout (MBW) and impulse oscillometry (IOS) and included spirometry and diffusing‐capacity test (DLCO). One‐minute‐sit‐to‐stand test estimated physical function, and breathing difficulties were defined as answering “yes” to the question “do you experience lingering breathing difficulties?” Spirometry, DLCO, and IOS were normal in almost all cases (spirometry: 90%, DLCO: 98%, IOS: 88%), while MBW identified ventilation inhomogeneity in 50%. Breathing difficulties (*n* = 21) was associated with increased MBW‐derived S_acin_. However, physical function did not correlate with SAD. Among individuals with breathing difficulties, 25% had reduced physical function, 25% had SAD, 35% had both, and 15% had normal lung function and physical function. Despite spirometry and DLCO being normal in almost all post‐COVID‐19 individuals, SAD was present in a high proportion and was associated with lingering breathing difficulties.

## 1 INTRODUCTION

In April 2023, the World Health Organization (WHO) estimated that there were over 755,000,000 survivors of COVID‐19 infection globally (World Health Organization, 2023). Lingering respiratory symptoms are common after both mild (Augustin et al., 2021) and severe (The PHOSP‐COVID Collaborative Group, 2022) acute infection, and persist in high proportions of both hospitalized and non‐hospitalized individuals for a long time after the acute phase (Jennings et al., 2021; The PHOSP‐COVID Collaborative Group, 2022). Studies on lung function following COVID‐19 have mostly focused on using spirometry to measure the function of the large to mid‐size airways (>2 mm internal diameter), but spirometry is often found to be within the normal range, even in patients who have had severe pneumonia findings on computed tomography (CT) (Frija‐Masson et al., 2020).

The small airways are often affected in acute severe COVID‐19, and postmortem studies in COVID‐19 deaths demonstrate a reduction of alveolar type II cells in samples collected from deceased patients (Melms et al., 2021). SARS‐CoV‐2 induces lung epithelial and endothelial injuries (Ackermann et al., 2020), contributing to impairment of peripheral lung function. Furthermore, the vascular involvement in SARS‐CoV‐2 (Guzik et al., 2020) may negatively affect gas exchange over the alveolar‐capillary membrane. The efficiency of gas exchange between the lung and pulmonary capillaries, measured by the diffusing capacity of the lung for carbon monoxide (DLCO), has been shown to be reduced following severe COVID‐19 (Björsell et al., 2023; Ekbom et al., 2021; González et al., 2021; Mo et al., 2020), especially in the presence of lung capillary injury (i.e., pulmonary embolism) (Calabrese et al., 2021). DLCO generally improves over time (Thomas et al., 2021), but one study found that in 33% of cases it remained abnormal 12 months after discharge (Wu et al., 2021). Nevertheless, DLCO impairment has been shown to be a poor predictor of perceived dyspnea (Froidure et al., 2021). Abnormalities in the pulmonary gas exchanging area following COVID‐19 have been demonstrated using ^129^xenon (Xe) magnetic resonance imaging (MRI), and the impairments were associated with breathlessness (Grist et al., 2021). However, the ^129^Xe MRI method is expensive and cumbersome, and the function of the small airways (i.e., ventilation distribution) can be assessed more conveniently and cost‐effectively using multiple breath washout (MBW). Small airway dysfunction (SAD) is a broadly defined concept, based on any of the methods available to assess pathology in small airways. It therefore includes different aspects of small airway functioning.

In the present study, we wanted to compare how different physiological methods reflect distinctive aspects of SAD following COVID‐19. In addition to MBW, we also assessed small airway function using impulse oscillometry (IOS), reflecting mechanical properties (i.e., resistance and reactance) in the lung, as a more clinically available tool for SAD assessment. Moreover, we wanted to examine the extent to which SAD is related to lingering breathing difficulties and physical function, as patients in the aftermath of COVID‐19 also seem to frequently report dyspnea at exertion (Abdallah et al., 2021). As SARS‐CoV‐2 specifically affects alveolar type‐II cells, we hypothesized that small airway function is affected in individuals with breathing difficulties following COVID‐19, and more specifically that the distribution of the ventilation in the acinar airways is disturbed.

## 2 MATERIALS AND METHODS

### 2.1 Study design and recruitment

This cross‐sectional study was based on data from 70 participants in an ongoing larger longitudinal cohort study performed at Sahlgrenska University Hospital, Gothenburg, Sweden. Individuals with a prior COVID‐19 infection (*n* = 58) were recruited via primary health care, a post‐COVID outpatient clinic at the Sahlgrenska University Hospital, and advertisements. Ten of the recruited cases were excluded: one due to pregnancy, one due to having a systemic inflammatory disease, two due to inability to perform the lung function tests, one due to a very short time since acute infection (14 days), and the remaining five due to a high likelihood of having been infected with the Omicron virus variant, as they had their initial infection after December 2021; this variant was very uncommon in Sweden until then. Healthy controls, comprising 22 non‐COVID‐19 infected individuals, were recruited via advertisement alone. Examination occurred between May 2021 and December 2022. The Ethics Committee in Sweden approved the study (ref: 2020‐05681), and all individuals gave written consent prior to participation.

### 2.2 Participants and study protocol

The COVID‐19 group comprised 48 participants aged 21–63 years (Table 1). COVID‐19 infection was verified by a positive SARS‐CoV‐2 ribonucleic acid (RNA) real‐time polymerase chain reaction (RT‐PCR) test via a nasal swab; if a PCR test was not performed at the time of acute infection, COVID‐19 was verified by presence of antibodies against SARS‐CoV‐2 in blood at the time of participation. Only individuals reporting respiratory impairment and/or cough in the acute phase and/or aftermath of disease were included. Patients requiring mechanical ventilation in the acute phase of the disease were excluded. Other exclusion criteria were uncontrolled cardiovascular disease, systemic inflammatory disease, and pregnancy.

**TABLE 1:** Demographic data in 70 participants with (*n* = 48) and without (*n* = 22) a prior COVID‐19 infection, for the total groups and divided by presence of breathing difficulties in the COVID‐19 group. Data are given as median (25th; 75th percentile) or proportion (%).

	Non‐COVID‐19 group, *n* = 22	COVID‐19 group, *n* = 48	Breathing difficulties, *n* = 21	No breathing difficulties, *n* = 27
Age (years)	54.5 (42.3; 59.2)	48.1 (39.4; 54.8)	51.9 (41.9; 58.3)	46.3 (33.6; 52.7)
Gender (male/female)	8/14	16/32	7/14	9/18
Height (cm)	168.0 (165.0; 176.5)	172.0 (166.1; 179.5)	174.0 (16.5; 180.0)	170.0 (165.5; 176.0)
Weight (kg)	69.2 (62.0; 85.6)	72.0 (66.9; 84.0)	81.0 (71.0; 84.5)	71.0 (63.0; 81.0)
BMI (kg/m^2^)	25.0 (23.3; 26.9)	24.9 (22.6; 26.1)	25.4 (23.8; 30.0)	24.1 (22.1; 26.1)
Ever‐smoker	0/22 (0%)	17/48 (35%)	10/21 (48%)	7/27 (26%)
Current smoker	0/22 (0%)	3/47 (6%)*	1/20 (5%)*	2/27 (7%)
Pack‐years	‐	0 (0; 4.5)*	0 (0; 5)*	0 (0; 0.5)
Asthma diagnosis	5/22 (23%)	11/48 (23%)	4/21 (19%)	7/27 (26%)
mMRC ≥2	0/22 (0%)	9/22 (41%)	7/21 (33%)	2/27 (7%)
Weeks between infection and inclusion	‐	65 (30; 87)	75 (46; 96)	65 (30; 82)
Hospitalized in the acute phase	‐	4/48 (8%)	2/21 (10%)	2/27 (7%)
Breathing difficulties in the acute phase	‐	36/48 (75%)	15/21 (71%)	21/27 (78%)
Cough in the acute phase	‐	35/48 (73%)	12/21 (57%)	23/27(85%)
Lingering breathing difficulties	‐	21/48(44%)	21/21 (100%)	0/27 (0%)
Lingering cough	‐	14/48 (29%)	8/21 (38%)	6/27(22%)

Abbreviations: BMI, body mass index; mMRC, modified Medical Research Council dyspnea scale.

* Data missing in one ever‐smoker.

The study protocol included IOS, spirometry, nitrogen (N_2_) MBW, DLCO, and the 1‐minute sit‐to‐stand test (1‐MSTST). IOS and spirometry were performed pre and post inhalation of 400 μg salbutamol (Airomir®, Teva Sweden AB, Sweden) via a chamber (Vortex®, PARI Medical GmbH, Germany). Questionnaires included were the modified Medical Research Council (mMRC) dyspnea scale (UK Research and Innovation, 2023) and an in‐house developed questionnaire assessing smoking history, disease severity, and presence of respiratory symptoms. All participants in the COVID‐19 group were also examined by a physician.

### 2.3 Lung function measurements

Spirometry, IOS, and DLCO were conducted using a Jaeger MasterScreen PFT system (Care Fusion, Germany) according to current guidelines (Graham et al., 2017, 2019; King et al., 2020). Indices reflecting small airway resistance (resistance at 5 Hz–resistance at 20 Hz; R5–R20) and reactance (area of reactance; AX) were derived from IOS, while DLCO and DLCO/alveolar volume (VA) were derived from the DLCO test. DLCO outcomes were corrected for current hemoglobin level.

N_2_ MBW was performed according to guidelines (Robinson et al., 2013) using an Exhalyzer D (Eco Medics AG, Switzerland) with version 3.3.1 of the Spiroware software package. Indices were derived to represent global ventilation heterogeneity (lung clearance index; LCI) as well as ventilation heterogeneity in the conducting airways (S_cond_) and the small airways at the entrance to the acinar zone (S_acin_). S_cond_ and S_acin_ were generated by concentration‐normalized phase III slope (Sn_III_) analysis. Differences in breathing pattern and lung size were corrected for by multiplying Sn_III_ by the expiratory tidal volume (VT) of each subsequent breath, before further calculations. This article therefore actually reports S_cond_ x VT and S_acin_ x VT, but these are denoted S_cond_ and S_acin_ for simplicity.

### 2.4 Questionnaires and definition of breathing difficulties among COVID‐19 individuals

All participants answered an in‐house developed questionnaire covering medical history, current and previous symptoms, smoking history, and medication. We also included the mMRC dyspnea scale to assess degree of breathlessness among both controls and COVID‐19 individuals. However, the definition of having lingering breathing difficulties following COVID‐19 infection is solely defined by answering “yes” to the question “Do you experience lingering breathing difficulties?” included in our in‐house questionnaire. Degree of severity in the acute COVID‐19 phase was assessed by the World Health Organization clinical progression scale (The Lancet Infectious Diseases, 2020), which classifies severity into five categories: uninfected, ambulatory mild disease, hospitalized (moderate disease), hospitalized (severe disease), and dead.

### 2.5 Physical function

Physical function was quantified using the 1‐MSTST, performed according to the recommendations summarized by Bohannon and Crouch (2019). In addition to these recommendations, heart rate (HR) and peripheral capillary oxygen saturation (SpO_2_) were registered immediately before and after the test. Number of cycles was related to findings in a population‐based reference cohort (Strassmann et al., 2013).

### 2.6 Statistics

Lung function and 1‐MSTST outcomes were treated as nonparametric data, and we conducted two group comparisons using the Mann–Whitney *U*‐test. These comparisons included control group versus the COVID‐19 group, COVID‐19 individuals with versus without lingering breathing difficulties, controls versus COVID‐19 individuals without breathing difficulties, as well as controls versus COVID‐19 individuals with breathing difficulties. A correlation matrix was included to demonstrate strength and direction of relationship between included lung function outcomes. Spearman's rank correlation coefficient (*ρ*) together with a Bonferroni‐corrected *p*‐value was reported in the matrix. Binary logistic regression was used to predict the probability of having breathing difficulties. A one‐way ANCOVA was conducted to determine if S_acin_ (*z*‐score) significantly differed between individuals with and without breathing difficulties after controlling for time since acute infection. A two‐sided *p* < 0.05 was considered statistically significant. Lung function outcomes are presented as *z*‐scores; for spirometry (Quanjer et al., 2012) and DLCO (Stanojevic et al., 2017) these were based on Global Lung function Initiative (GLI) reference equations, and for IOS and N_2_ MBW they were based on local reference values elicited from 158 and 400 healthy controls, respectively (Kjellberg et al., 2016). Upper and lower limits of normal (ULN and LLN) were defined as ±1.96 *z*‐score. Version 28 of SPSS Statistics (IBM) was used for the statistical analyses.

## 3 RESULTS

The study included 22 participants with no prior COVID‐19 infection and 48 with a prior COVID‐19 infection. In the COVID‐19 group, the majority had a mild acute infection (44/48, 92%), while 4/48 (8%) were hospitalized; three with moderate and one with severe disease according to the WHO clinical progression scale (The Lancet Infectious Diseases, 2020). However, none of the participants required mechanical ventilation in the acute phase. There was a large variation in time between infection and inclusion, with a range of 10–131 weeks and a median of 65 weeks (Table 1). Lingering breathing difficulties was reported by 21/48 (44%) participants.

### 3.1 Lung function

The traditionally used lung function tests, spirometry and DLCO, were within the normal range in almost all participants: 43/48 (90%) for spirometry and 47/48 (98%) for DLCO. Increased resistance in small airways, as determined by R5–R20, was present in 4/48 (8%). The reactance in small airways (AX) was also abnormal in these four participants and in an additional two participants, for a total of 6/48 (13%).

**FIGURE 1:**
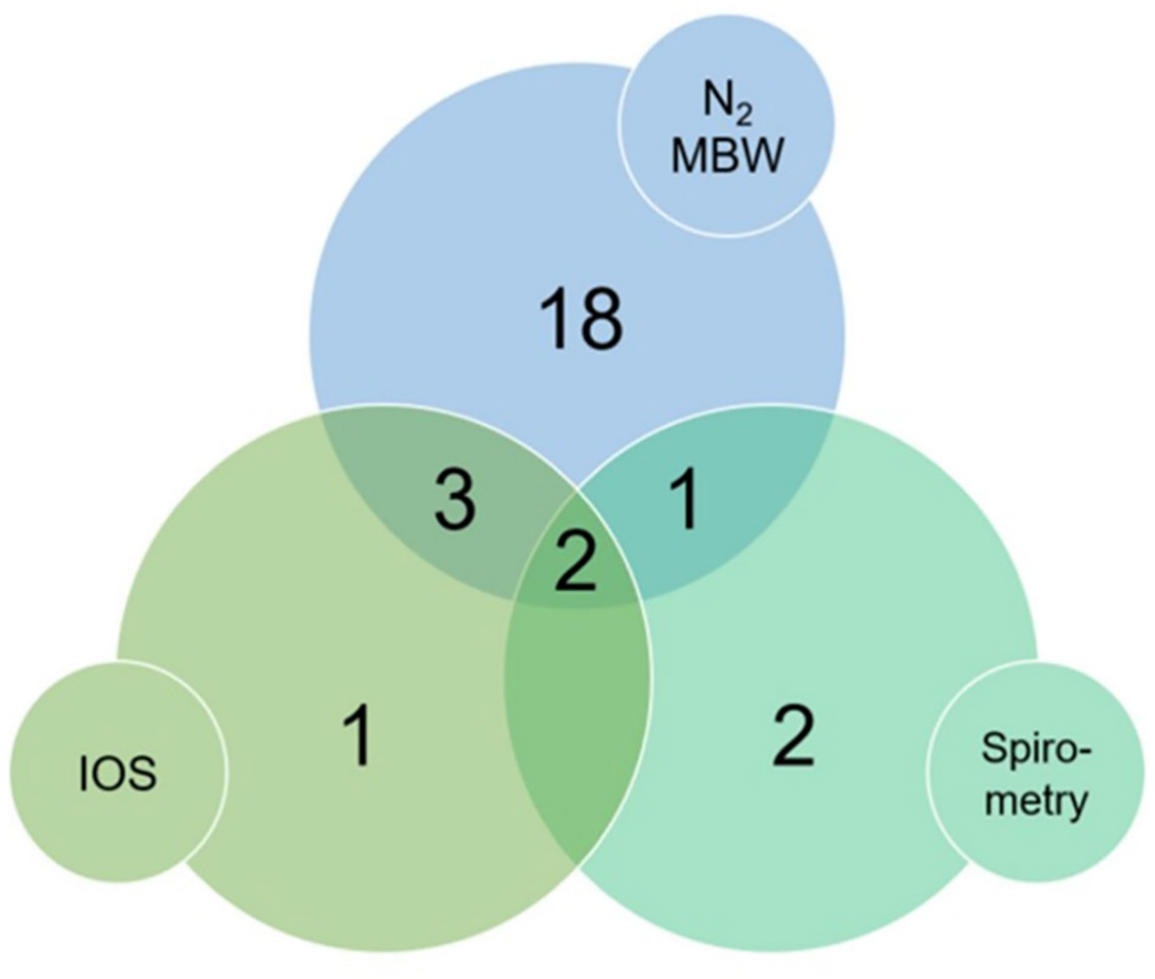
Concordance between abnormal findings across nitrogen multiple breath washout (N_2_ MBW), impulse oscillometry (IOS), and spirometry in 48 participants with a prior COVID‐19 infection.

MBW‐derived LCI was abnormal in 13/48 (27%) of participants, S_cond_ was abnormal in 8/48 (17%), and S_acin_ was abnormal in 12/48 (25%) (Table 2). Overall, 24/48 (50%) showed abnormality in at least one MBW outcome (Figure 1).

**TABLE 2:** Lung function outcomes and number of sit‐to‐stand cycles in 70 participants with (*n* = 48) and without (*n* = 22) a prior COVID‐19 infection, for the total groups and divided by presence of lingering breathing difficulties in the COVID‐19 group. Data are given as median (25th; 75th percentile) and proportion (%) with findings <−1.96 or > +1.96 *z*‐scores.

Lung function outcome	Non‐COVID‐19 group, *n* = 22	COVID‐19 group, *n* = 48	*p*‐value, non‐COVID‐19 vs. COVID‐19	Breathing difficulties, *n* = 21	No breathing difficulties, *n* = 27	*p*‐value, breathing difficulties vs. no breathing difficulties
FEV_1_ (*z*)	0.34 (−0.49; 1.09)	−0.04 (−0.59; 0.79)	0.234	−0.05 (−0.70; 0.48)	−0.03 (−0.41; 0.97)	0.355
FEV_1_ < −1.96 *z*	0/22 (0%)	2/48 (4%)	NA	2/21 (10%)	0/27 (0%)	NA
FVC (*z*)	0.72 (0.20; 1.47)	0.50 (−0.06; 0.87)	0.068	0.20 (−0.01; 0.72)	0.58 (−0.08; 1.00)	0.313
FVC < −1.96 *z*	0/22 (0%)	1/48 (2%)	NA	1/21 (5%)	0/27 (0%)	NA
FEV_1_/FVC (*z*)	−0.61 (−1.13; 0.01)	−0.56 (−1.28; 0.02)	0.909	−0.84 (−1.28; −0.07)	−0.36 (−1.37; 0.06)	0.618
FEV_1_/FVC < −1.96 *z*	1/22 (5%)	3/48 (6%)	0.775	0/21 (0%)	3/27 (11%)	NA
FEF_75_ (*z*)	−0.28 (−0.62; 0.41)	−0.46 (−1.18; 0.19)	0.229	−0.73 (−1.00; 0.15)	−0.29 (−1.33; 0.22)	0.568
FEF_75_ < −1.96 *z*	0/22 (0%)	0/48 (0%)	NA	0/21 (0%)	0/27 (0%)	NA
R5–R20 (*z*)	−0.98 (−1.23; 0.03)	−0.81 (−1.32; 0.16)	0.742	−0.73 (−1.42; 0.16)	−0.89 (−1.23; 0.16)	0.917
R5–R20 >1.96 *z*	0/22 (0%)	4/48 (8%)	NA	3/21 (14%)	1/27 (4%)	0.188
AX (*z*)	−0.50 (−0.67; 0.16)	−0.22 (−0.69; 0.45)	0.527	−0.26 (−0.71; 0.35)	−0.17 (−0.61; 0.49)	0.670
AX >1.96 *z*	3/22 (14%)	6/48 (13%)	0.895	4/21 (19%)	2/27 (7%)	0.226
LCI (*z*)	0.51 (−0.39; 1.70)	0.74 (−0.24; 2.14)	0.686	0.84 (0.23; 3.10)	0.48 (−0.33; 1.84)	0.167
LCI >1.96 *z*	5/22 (23%)	13/48 (27%)	0.699	7/21 (33%)	6/27 (22%)	0.390
S_cond_ (*z*)	0.05 (−0.68; 0.87)	−0.38 (−1.41; 1.29)	0.433	0.12 (−1.19; 1.45)	−0.53 (−1.54; 1.21)	0.603
S_cond_ >1.96 *z*	3/22 (14%)	8/48 (17%)	0.746	4/21 (19%)	4/27 (15%)	0.696
S_acin_ (*z*)	0.10 (−0.67; 1.40)	0.67 (−0.14; 2.01)	0.297	0.90 (0.28; 2.86)	0.21 (−0.48; 1.56)	0.013
S_acin_ >1.96 *z*	5/22 (23%)	12/48 (25%)	0.837	8/21 (38%)	4/27 (15%)	0.065
DLCO (*z*)	0.53 (0.16; 0.94)	0.11 (−0.46; 0.49)	0.004	0.03 (−0.60; 0.33)	0.23 (−0.43; 0.54)	0.388
DLCO < −1.96 *z*	0/22 (0%)	1/48 (2%)	NA	0/21 (0%)	1/27 (4%)	NA
VA (*z*)	0.38 (0.23; 0.81)	−0.05 (−0.39; 0.70)	0.044	−0.10 (−0.48; 0.32)	0.01 (−0.27; 1.01)	0.442
VA < −1.96 *z*	0/22 (0%)	0/48 (0%)	NA	0/21 (0%)	0/27 (0%)	NA
DLCO/VA (*z*)	0.13 (−0.55; 0.89)	−0.21 (−0.71; 0.34)	0.105	−0.11 (−0.57; 0.24)	−0.30 (−0.71; 0.48)	0.716
DLCO/VA < −1.96 *z*	0/22 (0%)	0/48 (0%)	NA	0/21 (0%)	0/27 (0%)	NA
1‐MSTST (no of cycles performed)	47 (38; 55)	34 (29; 41) *	<0.001	33 (26; 37)	38 (30; 43)	0.059
1‐MSTST (no of cycles performed) ≤25th percentile reference population (Strassman et al., 2013)	3/22 (14%)	22/46 (48%)	0.006	12/20 (60%)	10/26 (38%)	0.147

FEV_1_, forced expiratory volume in one second; FVC, forced vital capacity; FEF_75_, forced expiratory flow at 75% of exhaled FVC; R5–R20, resistance at 5–20 Hz; AX, area of reactance. LCI, lung clearance index; DLCO, diffusing capacity of the lung for carbon monoxide; VA, alveolar volume; 1‐MSTST, 1‐minute sit‐to‐stand test; NA, not applicable; z, z‐score.

* Missing in two participants.

One participant had both abnormal DLCO and abnormal MBW but spirometry and IOS findings within the normal range.

Both DLCO and VA were lower in the COVID‐19 group than in the non‐COVID‐19 group, with median (IQR) *z*‐scores as follows: 0.11 (−0.46; 0.49) versus 0.53 (0.16; 0.94), *p* = 0.004 for DLCO and −0.05 (−0.39; 0.70) versus 0.38 (0.23; 0.81), *p* = 0.044 for VA (Table 2).

To better understand how different lung function measures are associated after COVID‐19, as well as how they are distinct from each other, correlations were calculated for these outcomes expressed in *z*‐score (Table 3). Because of multiple correlations, we applied a Bonferroni‐corrected α‐value. Sixty‐six correlations resulted in a corrected α‐value of 0.00076. The only outcome significantly correlated with outcomes derived from another method was DLCO derived VA, which showed a strong correlation with FEV_1_ (*ρ* = 0.61, *p* < 0.001) and with FVC (*ρ* = 0.81, *p* < 0.001).

**TABLE 3:** Correlations between lung function outcomes expressed in *z*‐score among 48 individuals with a prior COVID‐19 infection.

Spearman's rho *p*‐value	FEV_1_										
FVC	**0.77** 1.3*10 ^**−10**^	FVC									
FEV_1_/FVC	**0.68** 1.4*10 ^**−7**^	0.09 0.530	FEV_1_ /FVC								
FEF_75_	**0.81** 2.0*10 ^**−12**^	0.35 0.015	**0.91** 1.7*10 ^**−19**^	FEF_75_							
R5–R20	−0.18 0.211	−0.27 0.068	−0.01 0.938	−0.08 0.599	R5‐R20						
AX	−0.35 0.016	−0.37 0.009	−0.11 0.470	−0.22 0.141	**0.89** 2.3*10 ^**−17**^	AX					
LCI	−0.22 0.133	−0.16 0.278	−0.19 0.188	−0.25 0.081	0.27 0.067	0.23 0.117	LCI				
S_cond_	0.06 0.691	0.14 0.353	−0.01 0.941	−0.03 0.818	0.14 0.334	0.09 0.564	0.26 0.076	S_cond_			
S_acin_	−0.01 0.958	0.03 0.856	−0.12 0.410	−0.10 0.482	0.18 0.226	0.16 0.268	0.31 0.033	−0.08 0.611	S_acin_		
DLCO	0.34 0.017	0.42 0.003	0.11 0.464	0.18 0.216	0.07 0.653	0.04 0.784	−0.22 0.141	0.12 0.408	0.11 0.464	DLCO	
DLCO/VA	−0.17 0.248	−0.34 0.018	0.12 0.432	0.01 0.973	0.18 0.223	0.24 0.103	0.12 0.433	0.04 0.790	0.20 0.181	**0.53** 1.0*10 ^**−4**^	DLCO/VA
VA	**0.61** 4.4*10 ^**−6**^	**0.81** 4.9*10 ^**−12**^	0.11 0.465	0.29 0.045	−0.20 0.164	−0.29 0.043	−0.27 0.064	0.00 0.989	−0.05 0.731	0.39 0.006	−0.45 0.001

Correlations that are significant using a Bonferroni‐corrected α‐value (0.00076) are bolded.

Abbreviations: FEV_1_, forced expiratory volume in one second; FVC, forced vital capacity; FEF_75_, forced expiratory flow when 75% of FVC is exhaled; R5–R20, resistance at 5–20 Hz; AX, area of reactance; LCI, lung clearance index; DLCO, diffusing capacity of the lungs for carbon monoxide; VA, alveolar volume.

### 3.2 Lung function, physical function, and lingering breathing difficulties

**FIGURE 2:**
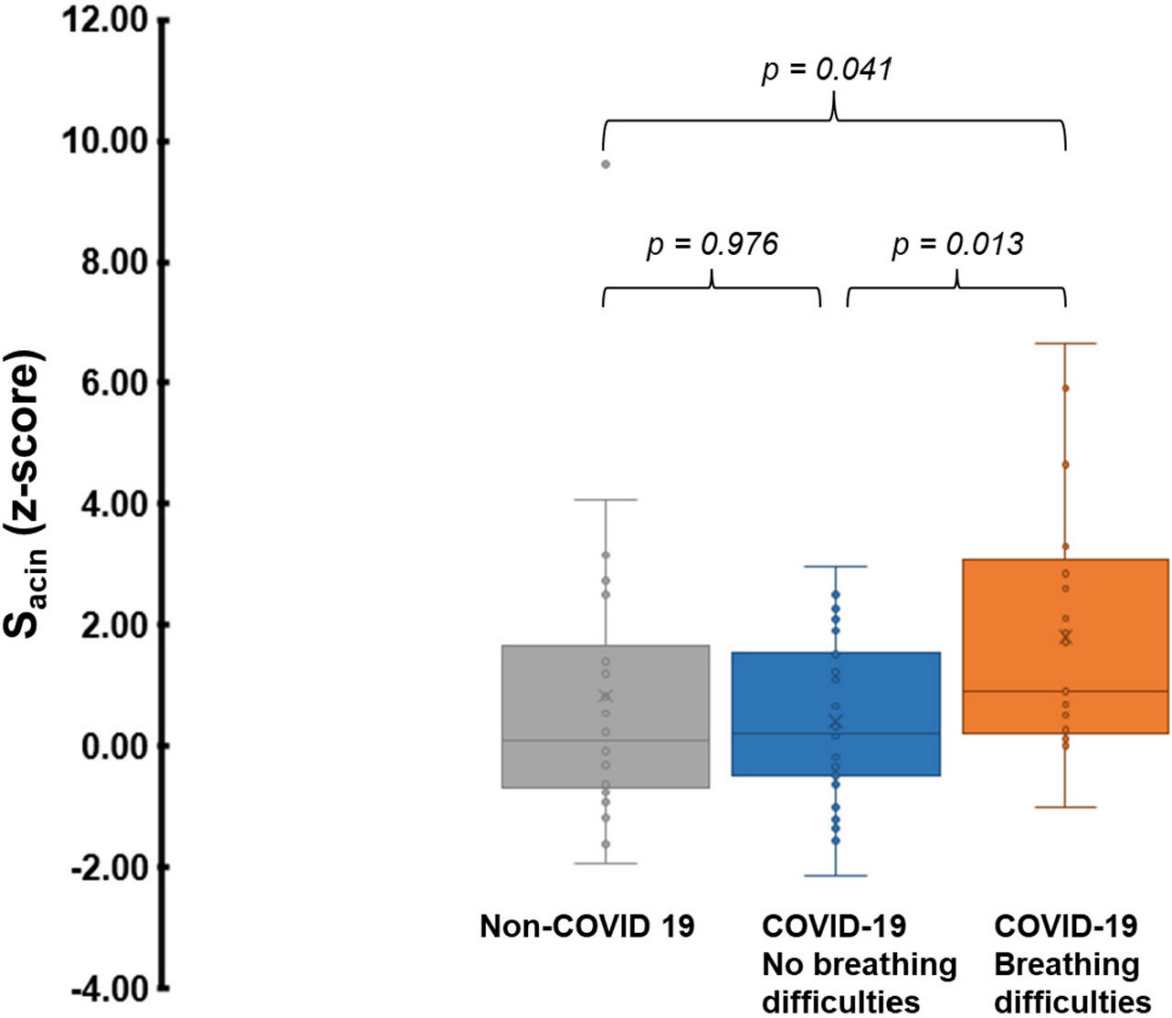
Acinar ventilation heterogeneity (S_acin_) in 22 non‐COVID‐19 participants and in 27 participants with no lingering breathing difficulties and in 21 participants with lingering breathing difficulties following COVID‐19. The lower and upper ends of each box represent the 1st and 3rd quartiles, and the horizontal line that divides the box is the median. The lower and upper whiskers represent the lowest and highest data points, excluding outliers. The average value is indicated by “x”, while individual data points are displayed as colored dots.

Participants reporting breathing difficulties had significantly higher S_acin_ (*z*‐score) compared to those without: median (25th; 75th percentile), 0.90 (0.28; 2.86) versus 0.21 (−0.48; 1.56), *p* = 0.013, and also compared to the non‐COVID‐19 group, 0.10 (−0.67; 1.40), *p* = 0.041 (Figure 2, Table 2). The difference in S_acin_ between individuals with and without breathing difficulties remained significant (*p* = 0.003) after controlling for time since acute infection. Moreover, in a logistic regression model including BMI, pack‐years smoked, S_acin_ (*z*‐score), and time since acute infection as independent variables, only S_acin_ showed a significant association with breathing difficulties (OR: 1.67, 95% confidence interval: 1.07–2.59, *p* = 0.02). No other lung function outcome differed between individuals with and without breathing difficulties (Table 2). Time elapsed between the acute infection and participation did not differ significantly between individuals with lingering breathing difficulties and those without: median weeks (IQR), 75 (46; 96) versus 65 (30; 82), *p* = 0.448.

**FIGURE 3:**
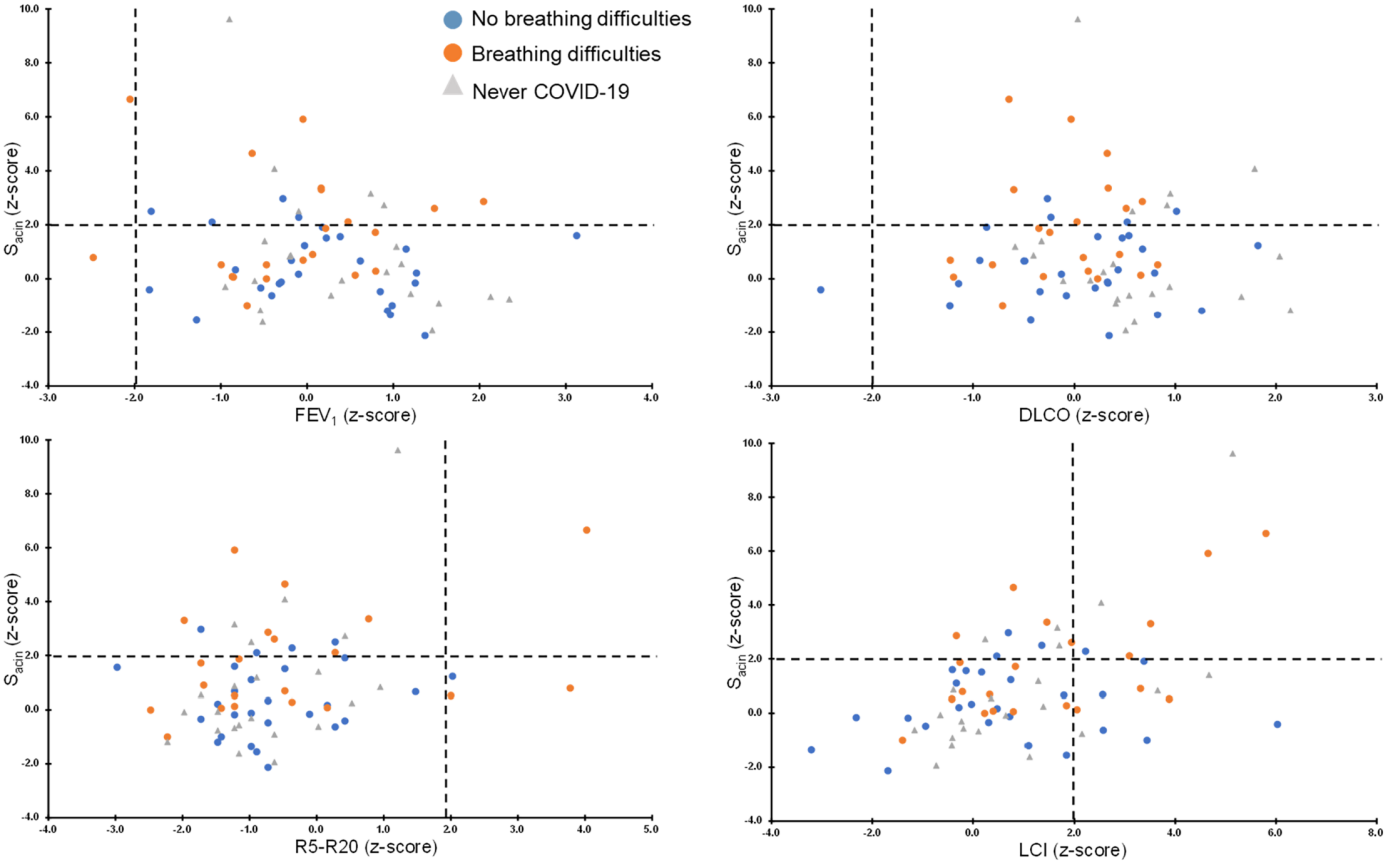
Relationship between acinar ventilation heterogeneity (S_acin_) and four other lung function outcomes in 48 participants with a prior COVID‐19 infection, separated by presence/absence of lingering breathing difficulties, and in 22 controls. Dashed lines denote the upper and lower limits of normality. DLCO, diffusing capacity of the lung for carbon monoxide; FEV_1_, forced expiratory volume in one second; LCI, lung clearance index; R5–R20, resistance at 5–20 Hz.

Although SAD, as, assessed by MBW was found in 12/21 (57%) of participants with breathing difficulties (four with both LCI and S_acin_ > ULN, three with only S_acin_ > ULN, two with both LCI and S_cond_ > ULN, one with both S_cond_ and S_acin_ > ULN, one with only S_cond_ > ULN, and one with only LCI > ULN), spirometry was abnormal in only 2/21 (10%) of these (one with both FEV_1_ and FVC < LLN, and one with only FEV_1_ < LLN) and DLCO was abnormal in none. Oscillometry identified SAD in 4/21 (19%) participants (one with only AX > ULN and three with both AX and R5‐R20 > ULN) (Figure 3). Among the symptom‐free participants, abnormal MBW outcomes were seen in 12/27 (44%; only LCI > ULN in four, only S_cond_ > ULN in three, only S_acin_ > ULN in three, both LCI and S_acin_ > ULN in one and both LCI and S_cond_ > ULN in one), and abnormal IOS was seen in another two (one with only AX >ULN and one with both R5‐R20 and AX > ULN).

Participants with lingering breathing difficulties performed fewer sit‐to‐stand cycles than those without, although the difference did not reach statistical significance: median (25th; 75th percentile), 33 (26; 37) versus 38 (30; 43), *p* = 0.059. These two groups did not show differences in SpO_2_ drop or heart rate increase directly after the test. Data for the 1‐MSTST were missing in two participants due to knee pain and technical issues.

The only lung function outcome to correlate with number of performed sit‐to‐stand cycles was VA (*z*‐score) (*ρ* = 0.38, *p* = 0.010). In total, 22/46 (48%) performed fewer cycles than the 25th percentile in a healthy reference population (26); this proportion was not significantly higher in the breathing difficulties group compared to the symptom‐free group, *p* = 0.147.

**FIGURE 4:**
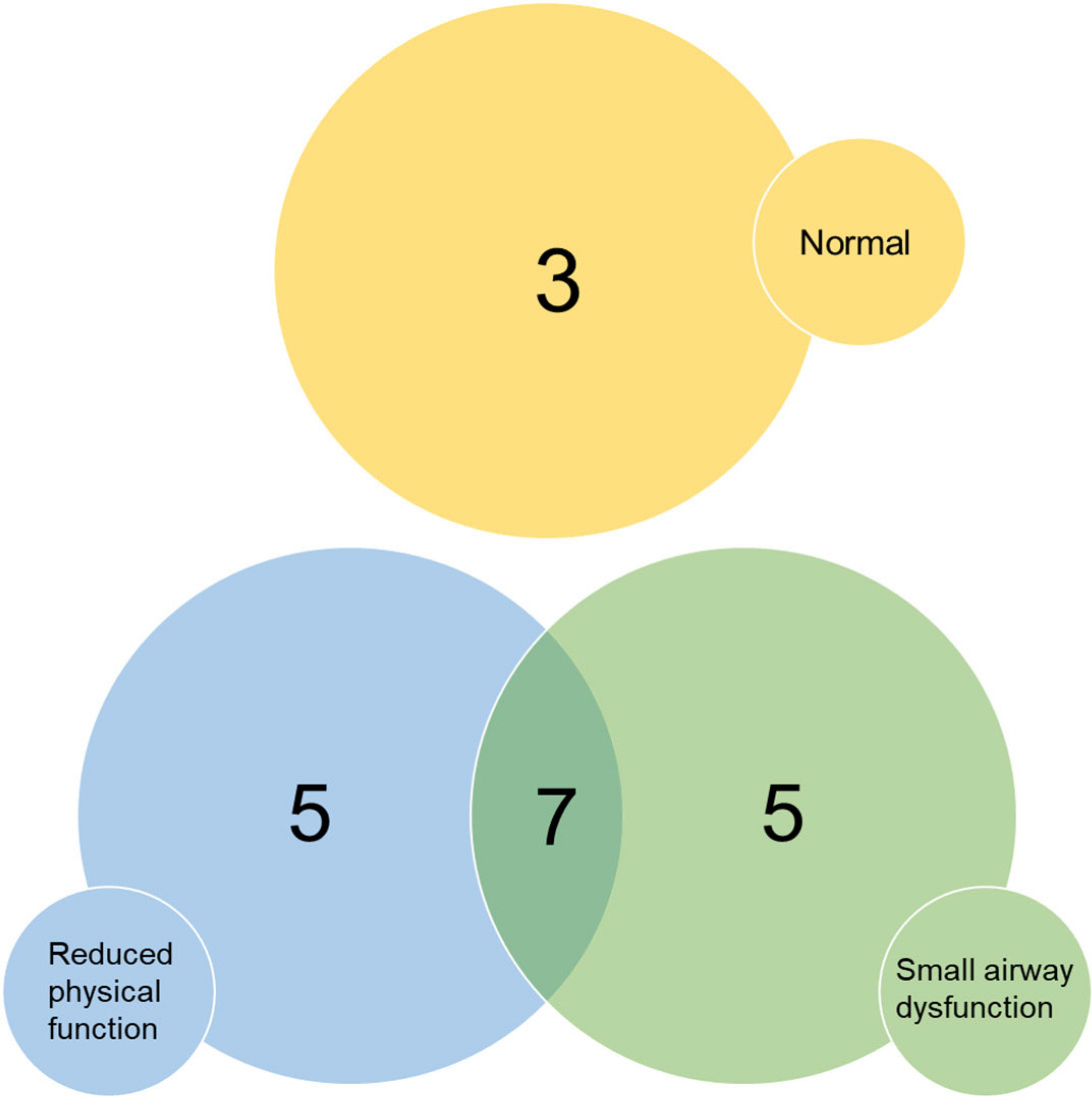
Among 20 participants with lingering breathing difficulties after COVID‐19 infection, five had reduced physical function as assessed by the 1‐minute sit‐to‐stand test, five had small airway dysfunction detected with multiple breath washout, seven had both of these conditions, and three had neither.

In summary, four subgroups could be identified among the 20 participants with breathing difficulties (1‐MSTST missing in one): Five participants had reduced physical function, five had SAD assessed by MBW, seven had both, and three had normal physical function and no signs of SAD (Figure 4).

## 4 DISCUSSION

To the best of our knowledge, this is one of the first studies to report outcomes from MBW reflecting ventilation distribution in the conducting (S_cond_) and acinar (S_acin_) lung compartments in individuals following COVID‐19. The participants were examined an average of 15 months after the acute phase, and most of them had experienced mild COVID‐19. Still, 44% reported lingering breathing difficulties, even though most spirometry and DLCO outcomes were within the normal range.

SAD as assessed by MBW was abnormal (in at least one MBW‐derived outcome) in half of the participants with a previous COVID‐19 infection, regardless having lingering breathing difficulties or not, while SAD assessed by IOS was within the normal range (both normal R5‐R20 and AX) in almost all (88%). S_acin_ was the only lung function outcome that was significantly increased in participants with lingering breathing difficulties. These findings are of clinical importance, as they may explain some of the remaining respiratory symptoms after COVID‐19. It is also noteworthy that traditional lung function measures were not sensitive enough to detect the pathology.

Among participants with breathing difficulties, the findings suggested the presence of subgroups with different explanations for the symptoms; 25% had reduced physical function, 25% had MBW‐detected SAD, and 35% had a combination of these, while 15% did not present with any physical or lung function impairment at all. The cause of breathing difficulties in the latter subgroup remains unknown.

The 1‐MSTST, a physical test that requires both good muscle strength and oxygenation at exertion, correlated only with alveolar volume among all reported lung function outcomes. We conclude that there are many causes behind lingering breathing difficulties in individuals post COVID‐19, and it seems of high importance to establish methods for their identification. In this cohort we also identified a group with SAD despite having no respiratory symptoms at all.

### 4.1 Definition of lingering breathing difficulties

All participants completed the mMRC dyspnea scale. No controls and 7 cases answered in the affirmative. The question “lingering breathing difficulties” identified however many more cases (*n* = 21). Although validated questionnaires are preferable, we decided to use the question on “lingering breathing difficulties” that was more sensitive and specifically reflected breathing difficulties attributed to COVID‐19. The question may induce an attribution bias—subjects may be prone to consider that their breathing problems are due to COVID‐19. On the other hand, in this case attribution is of less importance, as only show the association between current symptoms and physiological measures. We consider it more important to capture a broader range of respiratory symptoms, than reflected by the mMRC questionnaire.

### 4.2 Ventilation inhomogeneity in the post‐COVID‐19 lung

Little research attention has been paid to the degree of ventilation inhomogeneity following COVID‐19 infection, as assessed by multiple breath inert gas washout. No study has reported S_acin_, and only a few studies have reported LCI (Kooner et al., 2022, 2023; Stylemans et al., 2021). In line with our findings, Stylemans et al. (2021) reported LCI to be within the normal range at both 10 weeks and 6 months after acute infection, even though all of their participants had experienced severe COVID‐19. Conversely, in the studies by Kooner et al. (2022, 2023) including both initially hospitalized and non‐hospitalized participants, LCI findings at 3 and 15 months after the acute infection did not correlate with reported respiratory symptoms at the baseline investigation, and reduced burden of respiratory symptoms at follow‐up was not associated with LCI improvement over time.

Kooner et al. also measured ventilation distribution using ^129^Xe MRI scans, expressed as the ventilation defect percent (VDP). They identified a significant proportion with ventilation defects 3 months after infection, and although a significant improvement was seen between 3 months and 15 months post infection, participants with a prior COVID‐19 infection still had significantly worse VDP at follow‐up than those with no prior infection. Neither VDP at 3 or 15 months, nor improvement in VDP, was associated with respiratory symptoms. However, VDP at 3 months was correlated with improvement in physical function (measured as 6‐min walk distance). The two measures for assessing ventilation inhomogeneity (LCI and VDP) demonstrated a significant correlation at follow‐up (*ρ* = 0.59, *p* < 0.001), and improvement in VDP was significantly correlated with improvement in LCI (*ρ* = −0.39, *p* = 0.03).

The finding by Kooner et al., investigating post‐acute COVID‐19 syndrome at the same time after acute infection as in the present study (median 15 months), supports our finding of the presence of ventilation inhomogeneity in the post COVID‐19 lung which is not necessarily associated with respiratory symptoms.

^129^Xe MRI scans can also be used to assess gas exchange over the alveolar‐capillary membrane. This measure has been shown to be reduced in post COVID‐19 patients with unexplained dyspnea, regardless of whether they were hospitalized in the acute phase (Grist et al., 2022). Matheson et al. (2022) also reported a trend toward ^129^Xe MRI derived gas transfer capacity being lower in post‐COVID‐19 patients (*p* = 0.06) even though their spirometry and DLCO were on average within the normal range. The capacity of gas transfer was not significantly changed at follow‐up 14 months after acute infection (Matheson et al., 2023), indicating residual small airway impairment long after the acute infection.

While LCI assesses global ventilation inhomogeneity, S_acin_ is a measure of ventilation inhomogeneity at or near the entrance to the acinar airways (Robinson et al., 2009). An increase in S_acin_ is seen when structural asymmetries are present between lung units at the entrance to the acinar airways, resulting in impaired gas mixing in this area (Verbanck & Paiva, 1990). S_acin_ seems to sensitively detect peripheral lung injury associated with breathing difficulties after COVID‐19. It is reasonable to assume that SARS‐CoV‐2 induced destruction of alveolar cells and remodeling in extracellular matrix (Guizani et al., 2021) will result in structural acinar asymmetries, reflected by S_acin_. This alveolar epithelium injury is further supported by findings of reduced gas transfer ability in ^129^Xe MRI studies in this patient group (Grist et al., 2022; Matheson et al., 2022, 2023).

### 4.3 Spirometry, IOS, and DLCO findings

It is not easy to make comparisons with previous studies on spirometry, DLCO, and IOS after COVID‐19 and associations with respiratory symptoms. Outcomes are dependent on the inclusion criteria, applied reference values, and definition of symptoms. Froidure et al. (2021) reported dyspnea in 25% of their participants 3 months after severe COVID‐19 and DLCO ≤2 *z*‐score in 45%, with a low concordance between the findings. Lerum et al. (2021). Reported an even higher prevalence (54%) of dyspnea 3 months after hospital admission, spirometry within the normal range for the majority, and reduced DLCO in a quarter of participants. Conversely, Cortés‐Telles et al. (2021) reported reduced FVC, FEV_1_, and DLCO in individuals with dyspnea 30–90 days after mild, moderate, or severe infection. However, both spirometry and DLCO outcomes were within the normal range on a group level. One year after moderate–severe COVID‐19 disease, Centanaro et al. (2022) demonstrated a weak correlation between percentage of predicted DLCO and dyspnea score (*R* = −0.23, *p* = 0.006), while no spirometry outcomes predicted dyspnea. Nevertheless, in a study by Nakshbandi et al., both cough and dyspnea were associated with spirometry outcomes. The latter study used home monitoring of spirometry and respiratory symptoms during 6 months after admission to hospital due to COVID‐19. Over this period, FVC improved significantly but there were no improvements in either cough or dyspnea score (Nakshbandi et al., 2022).

Overall, results from follow‐ups of individuals with lingering respiratory symptoms after COVID‐19 including spirometry and DLCO are not conclusive but indicate that spirometry is insensitive in detecting lung function changes after COVID‐19 infection.

Our findings suggest that IOS is also less sensitive in detecting SAD following COVID‐19. This has been reported by others (Lindahl et al., 2021; Tamminen et al., 2022), though those results are also contradictory. Two Finnish studies, one among patients following severe COVID‐19 (Lindahl et al., 2021) and the other investigating patients with mild COVID‐19 (Tamminen et al., 2022), reported no evidence of SAD assessed by R5–R20 and AX. In the latter study, the prevalence of productive cough as well as the dyspnea scores decreased between acute infection and the 2‐month follow‐up, but R5–R20 and AX increased between the visits.

Huang et al., on the contrary, reported abnormal R5 in 14% of participants 30 days after hospital discharge, although those with milder disease had more pronounced abnormalities than those with severe disease (Huang et al., 2020). Lopes et al. (2021) reported abnormal oscillometry findings in 88% at 2 months and 71% at 5 months after CT‐confirmed COVID‐19 pneumonia, in individuals still reporting cough and/or dyspnea at the 2‐month follow‐up. The high prevalence of abnormal findings in that study may be explained by a more severe acute infection, a high proportion of obese individuals in the cohort (median BMI: 29), and differently applied cutoff values.

Overall, although it seems that some studies suggest an obstructive pattern with increased oscillometry findings after COVID‐19, there is a large variation in outcomes. Based on the findings in our study, MBW seems to be preferential to IOS when detecting SAD in this patient group.

### 4.4 Physical function and peripheral lung function

Our finding that 48% of patients performed fewer sit‐to‐stand cycles than the 25th percentile in a reference population is in line with findings by Johnsen et al. (2021) in both hospitalized and non‐hospitalized individuals. However, we found no correlation between small airway function and physical function, and although an overlap was seen among participants with lingering breathing difficulties regarding SAD and reduced physical function (Figure 4), the reduced physical function cannot directly be explained by lung function impairment. We speculate that a dysfunctional breathing pattern might also explain the reduced physical function that has been reported in COVID‐19 patients with remaining dyspnea (Frésard et al., 2022).

### 4.5 Strengths and limitations

The major strength of this study is the multidimensional assessment of both central and peripheral lung function in post‐COVID‐19 individuals using non‐invasive diagnostic methods. However, the study has some limitations. As part of a larger ongoing longitudinal study, it is limited by the small number of participants. The inclusion criteria may have biased the selection of participants, and so the study cannot be used to estimate prevalence of breathing difficulties and lung function abnormalities in the population. Moreover, the recruitment procedure may have contributed to a selection bias, as those with remaining respiratory symptoms may be more prone to participate, particularly as time elapses. However, this does not reduce the validity of our main finding; that is, that SAD was present in more than half of those with lingering breathing difficulties (60%, Figure 4), despite normal spirometry and DLCO findings in almost all of them.

The lack of pre‐COVID‐19 lung function data makes it difficult to ensure that COVID‐19 was the main cause of the observed abnormalities. The cohort included individuals with asthma diagnosed prior to the pandemic and individuals with a history of smoking. However, the prevalence of asthma was the same in individuals with and without lingering breathing difficulties. The probability that increased S_acin_ may be due to having a smoking history is low, considering (i) the low number of pack‐years smoked in the group, (ii) the difference in S_acin_ between the two symptom groups remained significant when individuals with a smoking history were excluded from the analysis (*p* = 0.044), and (iii) S_acin_ did not differ between ever‐smokers and never‐smokers (*p* = 0.923). Thus, increased S_acin_ in the present study seems not to be driven by previous tobacco smoking. Furthermore, S_acin_ in individuals with lingering breathing difficulties was significantly increased compared to the non‐COVID‐19 group (*p* = 0.044). Still, the importance of having asthma or having a smoking history on lung function post‐COVID‐19 needs to be further studied in larger cohorts, preferably with lung function data available prior to COVID‐19 infection.

Large differences in follow‐up times are another limitation, given that the consensus in the literature suggests that both symptoms and lung function generally show improvement over time following acute infection. Nevertheless, this seems not to be a major confounder in our cohort, as S_acin_ remained significantly increased in individuals with lingering breathing difficulties after controlling for time since acute infection, as well as the lack of a significant association in the logistic regression analysis between follow‐up times and the likelihood of experiencing breathing difficulties. Moreover, some of the participants have had COVID‐19 multiple times. However, since we did not inquire about this possibility at the beginning of the study period due to a lack of knowledge regarding the potential for reinfection, we cannot incorporate this aspect into our analyses. To note is that our study focuses exclusively on their first occurrence of the infection. Finally, what is regarded as abnormal is based on reference values, where the cutoff values may not be fully appropriate for the present population.

## 5 CONCLUSION

In this study of individuals following mostly mild COVID‐19 disease, ventilation heterogeneity as assessed by MBW was present in half of the cohort, despite spirometry, DLCO, and IOS being normal in almost all cases. Our hypothesis, that specifically the ventilation distribution of the acinar airways may be disturbed after COVID‐19, seems to be correct. The present results indicate that SAD is a common finding, and that very peripheral lung function impairment (increased S_acin_) in particular is associated with breathing difficulties. However, SAD and reduced physical function were both independently present in individuals with lingering breathing difficulties. Thus, breathing difficulties following COVID‐19 might have many differing aetiologies which need to be accurately investigated in order to identify suitable diagnostic methods and apply individualized treatment.

The long‐term importance and clinical relevance of SAD in individuals without respiratory symptoms both remain unclear; these aspects need to be studied longitudinally.

## FUNDING INFORMATION

This study was funded by grants from InterReg and the Swedish Heart Lung Foundation (#20210128).

## CONFLICT OF INTEREST STATEMENT

ACO is a chair holder and a board member of PExA AB (www.PEXA.se). None of the other authors have any conflicts of interest to disclose.

## ETHICS STATEMENT

The Ethics Committee in Sweden approved the study (ref: 2020‐05681), and all individuals gave written consent prior to participation.

## Data Availability

The data supporting the findings of this study are available on request to the corresponding author.
